# The Potential Harm of Cytomegalovirus Infection in Immunocompetent Critically Ill Children

**DOI:** 10.3389/fped.2018.00096

**Published:** 2018-04-10

**Authors:** Raidan Alyazidi, Srinivas Murthy, Jennifer A. Slyker, Soren Gantt

**Affiliations:** ^1^University of British Columbia and BC Children’s Hospital, Vancouver, BC, Canada; ^2^Department of Pediatrics, Faculty of Medicine, King Abdulaziz University, Jeddah, Saudi Arabia; ^3^University of Washington, Seattle, WA, United States

**Keywords:** cytomegalovirus, pediatric critical care, viremia, reactivation, immunocompetent, outcome, ganciclovir

## Abstract

Cytomegalovirus (CMV) is a ubiquitous infection that causes disease in congenitally infected children and immunocompromised patients. Although nearly all CMV infections remain latent and asymptomatic in immunologically normal individuals, numerous studies have found that systemic viral reactivation is common in immunocompetent critically ill adults, as measured by detection of CMV in the blood (viremia). Furthermore, CMV viremia is strongly correlated with adverse outcomes in the adult intensive care unit (ICU), including prolonged stay, duration of mechanical ventilation, and death. Increasing evidence, including from a randomized clinical trial of antiviral treatment, suggests that these effects of CMV may be causal. Therefore, interventions targeting CMV might improve outcomes in adult ICU patients. CMV may have an even greater impact on critically ill children, particularly in low and middle income countries (LMIC), where CMV is regularly acquired in early childhood, and where inpatient morbidity and mortality are inordinately high. However, to date, there are few data regarding the clinical relevance of CMV infection or viremia in immunocompetent critically ill children. We propose that CMV infection should be studied as a potential modifiable cause of disease in critically ill children, and that these studies be conducted in LMIC. Below, we briefly review the role of CMV in immunologically normal critically ill adults and children, outline age-dependent differences in CMV infection that may influence ICU outcomes, and describe an agenda for future research.

## Introduction

Cytomegalovirus (CMV) is a double-stranded DNA herpesvirus that infects most of the world’s population (1, 2). Natural transmission is mostly through contact with viral shedding in breast milk, saliva, urine, or genital fluids (3–5). Similar to other herpesviruses, acute CMV infection is associated with high levels of lytic viral replication and dissemination throughout the body, followed by the establishment of viral latency in long-lived cell types that is responsible for lifelong persistence of infection (6).

Cytomegalovirus infection is typically diagnosed by serology (1, 7, 8). In addition to CMV IgG, use of CMV IgM and serial IgG avidity testing may help determine the timing of infection in selected situations (7, 9, 10). However, in infants, CMV serologic testing can be confounded presence of maternal IgG acquired passively *in utero* (3, 8). Therefore, diagnosis in infants requires either seroconversion (a change from negative to positive serologic testing) or, more often, by the detection of CMV in blood, saliva, or urine (3, 8). Isolation of CMV in culture or detection of CMV DNA or antigen in patient samples reflects lytic viral replication and is termed “active” infection (1, 2). Active CMV infection in a chronically infected person may be due to either reactivation of latent virus or reinfection with an exogenous strain of CMV (1, 2, 11, 12).

Acute CMV infection in healthy children or adults is nearly always asymptomatic or limited to mild non-specific illness. Periodic mucosal CMV reactivation and viral shedding is not uncommon during chronic infection, but systemic replication or viremia (detection of CMV in blood) is rare in immunologically normal individuals (4, 13, 14). By contrast, CMV viremia and end-organ disease is common in congenitally infected children and immunocompromised patients (15–18). More recent findings also suggest that CMV might have pervasive negative impact on health through indirect effects on the immune system (19–21). In particular, CMV has increasingly been recognized as a potential cause of disease in immunologically normal adults with critical illness (22–25). However, despite the fact that young children generally control CMV infection poorly compared to adults, little is known about the role of CMV in immunocompetent critically ill pediatric patients. Below, we review the evidence for CMV causing adverse clinical outcomes in immunocompetent critically ill adults, and provide a rationale for analogous studies to examine the impact of CMV on critical illness in immunocompetent children.

## Systemic CMV Reactivation in Immunocompetent Critically Ill Adults

### Observational Studies Linking CMV Viremia With Adverse Adult Critical Care Outcomes

There have been more than 30 studies of immunologically normal, critically ill adult patients demonstrating an association between systemic CMV reactivation and worse outcomes in intensive care units (ICUs), as summarized in recent reviews (22–25). In these studies, CMV reactivation was defined as the detection of CMV in blood samples or, when feasible, in bronchoalveolar lavage (BAL) fluid, using CMV DNA PCR, antigen immunocytochemistry, or growth in shell vial culture. CMV viremia occurs in approximately 35% of seropositive adults following admission to the ICU. By contrast, fewer than 1% of adult blood donors with chronic CMV infection have detectable viremia (13, 14). CMV viremia and viral load are positively correlated with worse outcomes, including prolonged ICU stay, length of mechanical ventilation, secondary infections, and death. CMV viremia occurs over a wide spectrum of ICU admission diagnoses, such as infection, trauma, burns, and major surgery (26–35). In particular, patients with bacterial sepsis or pneumonia have been reported to have a higher likelihood of CMV viremia (28, 33, 36). The risk of CMV viremia is not clearly related to disease severity at the time of admission based on prognostic and organ dysfunction scores, and the onset of CMV viremia tends to precede or accompany clinical decline (37, 38), which support the possibility that CMV causes, rather than merely being a marker of, worse ICU outcomes in adults (23–25).

### Mechanisms by Which Critical Illness May Cause CMV Viremia

Critical illness may impair immune control of CMV replication normally mediated by cytotoxic T cells and natural killer (NK) cells (39–41). Adults with sepsis show depletion of splenic T cells and reduced T cell responsiveness (42). Functional NK cell differences have also been reported between patients with septic shock or systemic inflammatory response syndrome and healthy controls (43). Furthermore, among patients with septic shock, those with CMV reactivation were found to have reduced NK cell function (44).

**Figure 1:**
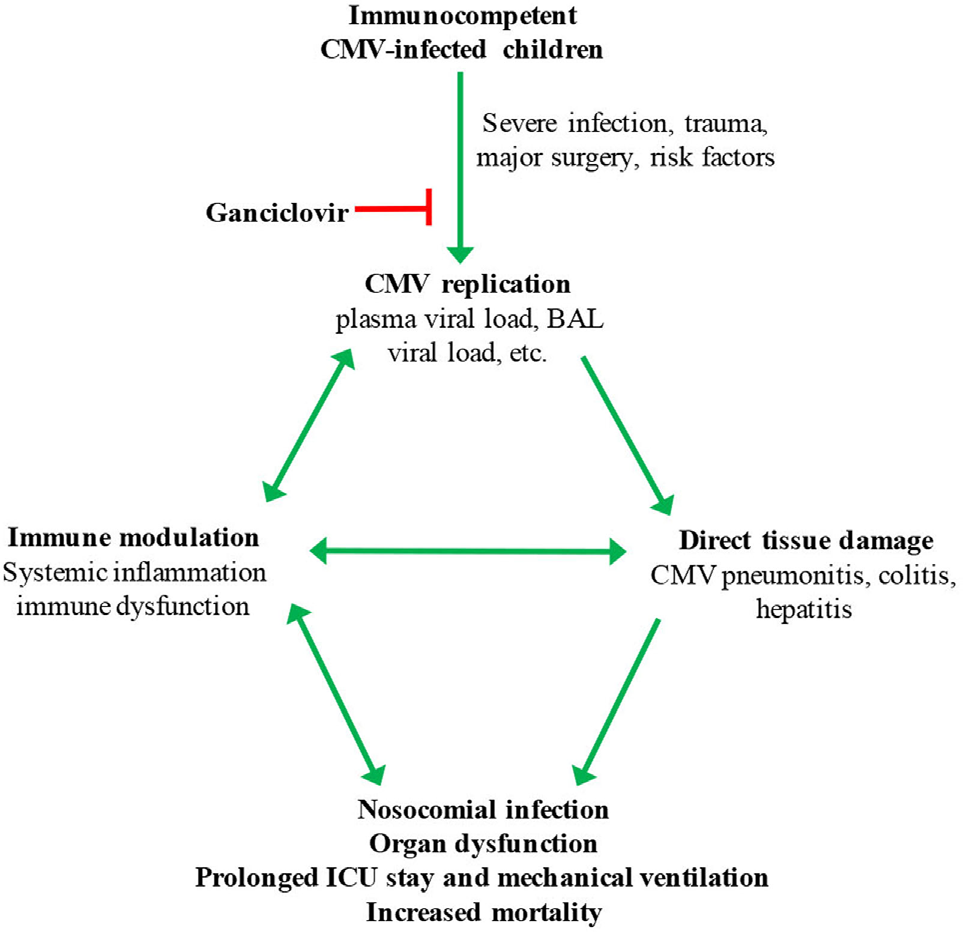
Hypothesized model for cytomegalovirus (CMV) reactivation in critically ill children. Possible risk factors for systemic CMV replication include severity of illness, pneumonia, malnutrition, blood transfusion, corticosteroid treatment, and young age. BAL, bronchoalveolar lavage.

Exposure of latently infected cells to a pro-inflammatory environment contributes to CMV reactivation (6). Thus, inflammation, e.g., due to trauma or sepsis, may initiate CMV reactivation in critically ill patients, and CMV replication then might in turn drive more inflammation in a feed-forward manner (Figure 1). CMV replication is associated with production of pro-inflammatory cytokines, including interleukin (IL)-1, IL-6, IL-8, and TNF-α (45–47). In a cohort of young adults, IL-6 levels were significantly higher in the sera of CMV seropositive compared to seronegative subjects (48, 49). Interestingly, CMV-infected adults were more likely to have a pro-inflammatory profile at 3 months after discharge from ICU, even after adjusting for illness severity and underlying conditions (50).

### Mechanisms by Which CMV Replication May Contribute to Clinical Decline

Adverse ICU outcomes often result from uncontrolled inflammation and/or impaired immunity (42). CMV infection may play a significant role in both of these processes through both direct and indirect mechanisms.

Cytomegalovirus replication in tissues of immunocompromised individuals is a well-recognized cause of end-organ damage, including pneumonitis, hepatitis, and colitis (11, 16–18). Although direct cytolytic effects of viral replication in tissues are central to the pathogenesis of CMV infection in the immunocompromised host, its role in immunocompetent critically ill individuals is relatively poorly defined. Some studies have reported frequent pulmonary CMV replication based on testing of BAL fluid and lung biopsies (26, 51). Histopathologic evidence of CMV colitis was also found in immunocompetent adult patients admitted to ICU for other causes (52). In addition, CMV has been reported to cause kidney and liver damage in immunocompetent adult ICU patients (53–55). Murine studies add further support to the hypothesis that CMV replication contributes to acute lung injury in adults with sepsis or severe trauma. Sepsis in immunocompetent mice causes murine CMV reactivation in the lungs, as well as pulmonary inflammation and fibrosis that is prevented by treatment with the antiviral drug ganciclovir (56, 57).

In transplant recipients, CMV is associated with the so-called “indirect” effects on the immune system, including increased risk of bacterial and fungal infections (52, 58, 59). Some studies suggest that CMV-induced immunomodulation may increase the risk of nosocomial infections in immunocompetent ICU patients (22, 53). These findings would seem to be consistent with our emerging understanding of the profound impact CMV infection has on the immune system generally (21, 60, 61).

### Interventional Studies of CMV Infection in Immunocompetent Critically Ill Adults

Randomized clinical trials exploring the effect of CMV treatment on outcomes in adult ICU patients are ongoing, and have the ability to determine the causality of CMV in these associations (22, 62). A recent open-label randomized trial was conducted among CMV seropositive, immunocompetent mechanically ventilated patients in a single ICU in the UK. Participants were assigned to receive prophylactic low-dose valganciclovir, high-dose valacyclovir, or no treatment (63). The primary outcome was time to CMV viremia by PCR. As expected, the antiviral groups were less likely to develop viremia (8 versus 35% in controls). This study demonstrates the efficacy of prophylactic antiviral use to prevent CMV viremia in ICU patients. However, the study was not powered to measure differences in clinical outcomes. Of note, the valacyclovir arm was discontinued early due to higher mortality, though all deaths were judged to be expected and attributable to underlying disease rather than any adverse antiviral effects.

Subsequently, a phase 2, randomized controlled multicenter trial of prophylactic ganciclovir or valganciclovir versus placebo in immunocompetent CMV seropositive adult ICU patients with respiratory failure and severe sepsis or trauma was published (64). The study failed to detect a difference in the primary outcome, blood IL-6 level, which was chosen based on the reported association of elevated IL-6 with ICU mortality and ventilator-associated lung injury (65, 66). However, in addition to reduced viremia, subjects in the antiviral treatment arm had significantly more ventilation-free days, a predetermined secondary outcome. This effect was particularly marked in the subgroup admitted for sepsis. A composite end point of mortality and increased serum IL-6 by 50% or >7 days ventilation was significantly higher in placebo group, and there was a trend toward increased mortality in the placebo group. Notably, antiviral treatment was not associated with significant side effects. This study provides the strongest evidence to date for a causal association between CMV viremia and worse outcomes in and provides preliminary support for the possibility that antiviral treatment may be beneficial for selected immunocompetent adult ICU patients (62).

## The Potential Role of CMV Infection in Immunocompetent Critically Ill Children

### Age-Dependent Differences in CMV Pathogenesis

There are qualitative and quantitative age-dependent differences in the immune responses of young children that impair the control of many viral infections, including CMV (67–69). Children with congenital CMV infection are most often asymptomatic, but commonly present with central nervous system disease, hepatitis, hematologic abnormalities, and pneumonitis. Sensorineural hearing loss and neurodevelopmental delay develop in approximately 20% of children with congenital CMV infection (15). Postnatal CMV infection rarely causes serious illness, though extremely premature infants can develop sepsis and severe end-organ disease (70–72). Even in the absence of CMV-related disease, compared with adults primary infection, children have substantially higher levels of viral replication as well as prolonged viremia and viral shedding in saliva or urine (4, 73). Though viral replication gradually declines over time, viral shedding during chronic infection remains substantially greater in children compared to adults (4, 73). Thus, as a result of more recent infection and differences in antiviral immunity, critically ill children might be expected to have worse control of CMV replication than adults.

### CMV Infection in Critically Ill Children

There are very few data available about the frequency and effects of active CMV infection in critically ill, immunocompetent children. We performed a pilot study of CMV infection in children admitted to the ICU of BC Children’s Hospital in Vancouver, Canada (unpublished data). Among the 27 children enrolled, the median age was 4 years (range 4 months–17 years). Only one child was CMV seropositive, and none were found to have viremia. This is consistent with the relatively low CMV seroprevalence among children in most high-income countries (74–77). By contrast, a study of children between 3 weeks and 2 years old hospitalized in Zambia found that 34% of HIV-uninfected children had CMV viremia (78). Of note, the prevalence of malnutrition was 36%, and being underweight was significantly associated with CMV viremia. In a retrospective study of Ugandan children 6 months–13 years old treated as outpatients for uncomplicated malaria, 11% had CMV viremia (79). Thus, even in the setting of moderate illness, systemic CMV replication is common in African children. In a prospective study that included 53 HIV-uninfected South African children ≤2 years old admitted to ICU with severe pneumonia, 12 (23%) were defined as having CMV pneumonitis based on DNA detection in BAL fluid and other clinical characteristics (80). Many of these children were treated with ganciclovir, but the published data are insufficient to determine whether disseminated CMV or antiviral treatment in HIV-uninfected children were associated with clinical outcomes (80).

## Determining the Impact of CMV Infection on Pediatric ICU Outcomes

### General Considerations

Well-designed cohort studies are needed to understand the role of CMV in immunocompetent critically ill children, to estimate the potential benefits, and to inform the design of appropriate pediatric treatment trials. Given the differences between children and adults in the natural history and outcomes of CMV infection, extrapolation from adult studies is inappropriate. As an example, in the adult studies conducted to date, the clinical impact of CMV has been ascribed to reactivation and dissemination of chronic infection (23–25). However, CMV viremia in pediatric ICU patients might often represent systemic viral replication related to recent infection rather than reactivation. To distinguish between reactivation of chronic infection and exacerbation of recent infection would be challenging, but might provide insights into age-dependent differences should they exist. Thus, in pediatrics studies, it would be more appropriate to explicitly evaluate the role of disseminated CMV replication as opposed to reactivation of chronic infection.

### Measuring CMV Replication in Pediatric ICU Patients

Quantitative CMV DNA PCR assays can reliably detect ≤50 international units/mL and have largely replaced older methods of detection (81, 82). Because high level CMV replication in saliva and urine is so prevalent in young children (73, 83), these measures are unlikely to be clinically meaningful for critically ill children except for confirming infection in infants with maternal antibody and concern for false-positive serology. As in adult studies, CMV replication in blood is likely to be the best measure of uncontrolled systemic viral infection in critically ill children. Although CMV in BAL fluid may simply represent shedding, high viral loads are predictive of CMV pneumonitis in transplant recipients and, perhaps, in critically ill children (80, 84, 85). Thus, pediatric ICU studies should incorporate CMV testing of BAL fluid, should it be collected for clinical purposes. In addition, detection of CMV in tissue biopsies (e.g., liver, colon), particularly by histopathology indicates invasive disease, and these specimens should be tested if available (Table 1).

**Table 1:** Suggested metrics for studies of cytomegalovirus (CMV) viremia in immunocompetent pediatric intensive care unit (ICU) patients.

CMV manifestations	Potential effects	CMV measures	Clinical outcome measures
Systemic inflammatory response syndrome	Multiorgan dysfunction	IgG, IgM, and urine PCR to diagnose infection	Mortality at 7, 14, and 28 days Ventilator-free days at 28 days
Pneumonitis Hepatitis	Prolonged ventilation Transaminitis, hyperbilirubinemia, impairment of synthetic liver function	Plasma/serum viral loadIf collected for clinical care: – BAL viral load – Histopathology (biopsies of lung, colon, liver, bone marrow)	Length of ICU/hospital stay Health-related quality of life at 28 days Organ dysfunction scores (PLEOD, SOFA, NPMOD, etc.)
Colitis	Enteric dysfunction, growth failure, nutritional deficiencies Nosocomial infection Blood product transfusions		Weight to height Z score Post-discharge mortality or readmission at 6 months Inflammatory markers in blood, BAL, and stool Blood transfusions Nosocomial infections Antibiotic use
Myelosuppression, neutropenia, thrombocytopenia, anemia			

*BAL, broncheoalveolar lavage; PLEOD, Pediatric Logistic Organ Dysfunction score; SOFA, Sequential Organ Failure Assessment score; NPMOD, New/Progressive Multiorgan Dysfunction*.

The risk of CMV disease is correlated to the level of viremia in many clinical scenarios. For example, in HIV-infected individuals and transplant recipients, CMV viral load is proportionally associated with progression of disease (86, 87). However, the threshold of CMV replication that is clinically relevant or that might warrant intervention depends on the patient population. For example, antiviral treatment of newborns with symptomatic congenital CMV infection is indicated, regardless of viral load, to reduce the risk of hearing loss and neurocognitive delay (8, 88–90). Selected transplant patients are monitored using CMV blood PCR in order to trigger preemptive antiviral treatment before the onset of disease, but the viral load threshold used varies based on immunologic status and other clinical criteria (87, 91, 92). The antiviral studies performed so far in adults have randomized all CMV seropositive ICU patients with the intention of preventing, rather than treating, viremia (63, 64). The optimal approach to treating CMV infection in pediatric ICU patients would depend on the associations and dose–response that are found, but might conceivably utilize risk-stratification based on age, underlying illness, viral load, or other factors.

### Pediatric Populations of Greatest Interest

Studying the impact of CMV infection on critically ill immunocompetent children is anticipated to be both easier and more clinically relevant in low- and middle-income countries (LMIC), where CMV infection is holoendemic and mortality and morbidity rates from sepsis are often dismal. In LMIC, nearly all children acquire CMV infection within the first few years of life (74). By contrast, the national US survey found that while CMV prevalence increases linearly with age, it reaches only ~50% by 30 years of age (75, 76).

In addition to the relatively low rates of CMV infection in North American and European children, the excellent outcomes of pediatric ICU patients there hinder the ability to detect a clinically important impact of CMV infection on critically ill children (93–96). Furthermore, strategies to reduce mortality in critically ill children in LMIC are urgently needed. For example, in the Kenyatta National Hospital pediatric ICU in Nairobi, Kenya, overall mortality is 42% (unpublished data). Similarly, pediatric ICU mortality rates of 25–50% have been reported in other LMICs in Africa and Asia (97–100). Other outcomes of interest such as length of stay and need for invasive ventilation are also frequently higher in LMICs (99, 100). In Siriraj Hospital, one of the largest hospital in Thailand, median length of ICU stay and rate of mechanical ventilation are 4–5 days and 64%, respectively (Dr. K. Lapphra, personal communication), which are more than double those in the BC Children’s Hospital ICU. Moreover, infections and sepsis account for most of pediatric ICU admission diagnoses in LMIC, which might again predispose to disseminated CMV infection and viremia (97, 98).

### Outcome Measure Considerations

Importantly, appropriate studies in children require the incorporation of pediatric-specific illness severity scores and outcome measures (Table 1). Mortality and morbidity in pediatric patients are predicted by progressive or new organ dysfunction, using validated scores (101–103). Evaluation of mechanical ventilation outcomes should include invasive as well as non-invasive modalities, which are now commonly used in children (94). A longer follow-up duration that used in adults studies should also be considered, given the high rate of readmission and post-discharge mortality in children of African and Asian countries (104–106). Higher rates of CMV viremia as well as worse post-discharge outcomes have been observed in underweight children (78, 106). Therefore, including nutritional assessments in pediatric studies is also essential.

## Conclusion

Strong evidence supports the association between CMV viremia and adverse outcomes in adult ICU patients (23–25), and early-phase interventional trial results suggest that antivirals in this population might be beneficial (62–64). Unfortunately, there are no studies yet in children that have adequately addressed this question. Not only is there an ethical obligation to include children in research according to the principle of justice (107–109), but we hypothesize that active systemic CMV infection might have an even greater negative impact on ICU outcomes in children than adults. Therefore, we propose that prospective observational studies should be conducted to characterize the association between CMV viremia and clinical outcomes in critically ill children, using metrics appropriate for pediatric populations. Furthermore, we suggest that such studies would best be performed in LMICs settings. Importantly, if CMV treatment were found to be beneficial for immunocompetent critically ill children, resource limitations would not preclude its implementation (110). If successful, studies in LMICs could result in reverse innovation to help targeting specific subpopulations of patients in high-income settings that might also benefit from CMV treatment. Studies in both adults and children should attempt to identify the mechanistic basis for the relationship between CMV and critical illness, and develop novel therapies to improve outcomes of critically ill patients.

## Ethics Statement

The pilot study Reactivation of Cytomegalovirus in Pediatrics (ReCIPe) for the prevalence of CMV viremia among pediatric critical care patients in BC Children’s Hospital was approved by BCCHR Ethics Board.

## Author Contributions

RA: literature review and writing; SG: reviewing, editing, writing, and knowledge check; JS: reviewing statistics and literature, editing, and writing; SM: checking literature and ICU data.

## Conflict of Interest Statement

The authors declare that the research was conducted in the absence of any commercial or financial relationships that could be construed as a potential conflict of interest.
